# Cancer in the offspring of female radiation workers: a record linkage study

**DOI:** 10.1038/sj.bjc.6604841

**Published:** 2009-01-06

**Authors:** K J Bunch, C R Muirhead, G J Draper, N Hunter, G M Kendall, J A O'Hagan, M A Phillipson, T J Vincent, W Zhang

**Affiliations:** 1 University of Oxford, Childhood Cancer Research Group, 57 Woodstock Road, Oxford OX2 6HJ, UK; 2 Radiation Protection Division, Centre for Radiation, Chemical and Environmental Hazards, Health Protection Agency, Chilton, Didcot, Oxfordshire OX11 0RQ, UK

**Keywords:** childhood cancer, *in utero* exposure, ionising radiation, occupational exposure, preconception irradiation

## Abstract

This study uses record linkage between the National Registry of Childhood Tumours (NRCT) and the National Registry for Radiation Workers to re-assess our earlier finding that the offspring of women radiation workers exposed to ionising radiation before the child's conception may be at an increased risk of childhood cancer. An additional 16 964 childhood cancer patients taken from the NRCT, together with the same number of matched controls, are included. Pooled analyses, based on the new and original datasets, include 52 612 cases and their matched controls. Relative risks (RRs) for maternal employment as a radiation worker, maternal exposure or not during the relevant pregnancy and pattern of employment relative to conception and diagnosis dates were calculated.

The new data provide no evidence of an increased risk of childhood cancer associated with maternal preconception radiation work and thus do not support our earlier finding of a raised risk in the offspring of female radiation workers. Considering the pooled data, a weak association was found between maternal radiation work during pregnancy and childhood cancer in offspring although the evidence is limited by the small numbers of linked cases and controls.

An earlier study (Draper *et al*, 1997a, 1997b; Sorahan *et al*, 2003) showed a statistically significant raised risk for childhood cancers among the children of exposed women radiation workers, but without any evidence of a dose–response relationship. Moreover, the effect was not confined to any specific childhood cancer diagnostic subgroup. This finding was based on small numbers of exposed cases (15) and controls (3).

This study re-examines the question of whether there is an association between maternal occupational exposure to ionising radiation and childhood cancer in subsequent offspring using data additional to those available earlier. In particular, it includes childhood cancer incidence data accrued within the National Registry of Childhood Tumours (NRCT) between 1987 and 1999. Analyses of these more recent data are compared with the findings obtained earlier.

## Methods

To assess the possible risks from maternal radiation exposure, we needed first to identify cases of childhood cancer diagnosed in the relevant period and to select matched controls. Record linkage to the National Registry for Radiation Workers (NRRW) was then used to ascertain which of the mothers of these case and control children had been occupationally exposed to ionising radiation.

Cases of childhood cancer (diagnosed before the child's fifteenth birthday) were identified from the NRCT (Stiller, 2007) and details of women radiation workers for inclusion in the linkage study were extracted from the NRRW (Muirhead *et al*, 1999). These registers, and the records from them included in this study, are described in more detail in the fuller Health Protection Agency (HPA) report (Muirhead *et al*, 2009).

Children eligible for our earlier study (Draper *et al*, 1997a, 1997b; Sorahan *et al*, 2003) were those born and diagnosed with cancer in Britain between 1952 and 1986. The current study additionally includes all British-born children diagnosed in Britain in the years 1987–1999. Ascertainment is considered virtually complete for leukaemia for this period and only slightly less so for other diagnostic groups (Stiller, 2007).

For each case child, the Office for National Statistics (ONS) was asked to locate the child's birth registration entry and select a control from the same birth register, matched on sex and born within 6 months of the case. For both case and control children, ONS returned birth registration details, including mother's name. For children born in Scotland, the corresponding information for both cases and controls was obtained from the General Register Office for Scotland, GRO(S).

Of the NRCT cases for the relevant period, birth registrations were obtained for some 93%, the remaining cases being born abroad, adopted or untraced. The record linkage that followed included the mothers of 16 964 case children and 16 964 matched controls.

Women in the NRRW were made aware of their option to withdraw from the study and nine women chose to do so. After excluding them, 15 840 female workers were included for possible linkage to the group of case and control mothers. The women included in the NRRW cohort had been employed, as radiation workers, at any time before 1 January 2000. In comparison to the earlier study, data for an extra 4200 female radiation workers were included. This includes both those radiation workers joining the participating organisations in more recent years and groups of earlier workers whose records were not available earlier.

Computerised record linkage was used to compare the names of the mothers of cases and controls in the study with those of the women radiation workers on the NRRW. A large number of possible links were generated and a series of checks was then applied to identify and exclude links where additional information (e.g. maternal birthplace) was in conflict. The record linkage process was undertaken entirely blind to the case/control status of the mothers involved, to avoid any bias. The record linkage methodology and validation process are described in detail in the HPA report (Muirhead *et al*, 2009).

Radiation doses are stored on the NRRW as annual totals. For the purposes of this study, in order to estimate doses before and around the time of conception or birth, doses were required for periods shorter than a year. Therefore, for those workers identified as mothers of childhood cancer cases or controls, more detailed dose information was sought from employing organisations together with information about monitoring for exposures from internal emitters.

### Statistical analyses

Statistical analysis involved the calculation of relative risks (RRs) to measure the association between maternal radiation exposure and the risk of childhood cancer – either taken as a whole or for specific types of cancer – for the offspring of various groups of workers as compared with the unexposed female population. Four maternal preconception dose categories and four *in utero* exposure categories were studied (see Table 1), the same categories as used earlier (Draper *et al*, 1997a, 1997b; Sorahan *et al*, 2003).

Relative risks of cancer in offspring were estimated separately for female workers monitored for internal radiation exposure and other radiation workers. Further analyses were performed to examine potential differences in cancer risk among offspring according to the timing of the mother's employment at an NRRW participating facility relative to the child's conception and cancer diagnosis.

Statistical analyses were performed using LogXact (2005), all statistical tests were two-sided and *P*-values<0.05 were taken as statistically significant. Further technical details relating to the statistical analyses can be found in the HPA report (Muirhead *et al*, 2009).

## Results

The new data include information on the mothers of 16 964 cases and their 16 964 matched controls. Among these, the mothers of four cases and seven controls were identified as being occupationally exposed before the child's conception. When combined with the earlier results, mothers of 52 612 childhood cancer cases and the same number of controls are included in the pooled analysis with 19 case mothers and 10 control mothers identified as being occupationally exposed.

Table 1 gives the results of analyses for maternal preconception dose, *in utero* dose and status of exposure to internal emitters (monitored/not monitored). Results are presented separately for the original data, the new data and the original and new data pooled. Most of the RRs for the new data shown in Table 1 are less than unity, and all of the confidence intervals include unity.

Overall, the new data provide no evidence of an association between childhood cancer and maternal preconception radiation work, in contrast to the increased risk for childhood cancer overall and ‘cancers other than LNHL’ found in the original data. When the new and original data are pooled there is no statistically significant increase in risk for cancer overall or for either diagnostic subgroup (Table 1). In particular, in the pooled data, the RR of all childhood cancers combined among the offspring of female radiation workers is 1.90 (95% CI: 0.84–4.58) based on 19 cases and 10 controls.

Among the group of children with an *in utero* dose due to maternal radiation work, there are no statistically significant raised risks for LNHL, ‘cancers other than LNHL’ or all childhood cancers combined in either the new or the original data taken alone (Table 1). However, when all childhood cancers are combined, there is some indication of a raised risk in the pooled data (RR 7.00, 95% CI: 0.90–315, based on seven exposed cases and one exposed control), a tendency found in both the old and new studies, though the numbers are very small (case/control ratios 4 : 0 and 3 : 1, respectively). There is no evidence of any association between the level of *in utero* dose and risk but such an association would be hard to detect, given the small numbers involved. There were no significantly raised risks for the offspring of female workers monitored for internal exposure.

Analyses were also performed to examine potential differences in cancer risk for offspring in relation to maternal employment pattern. Details of the exposure periods considered are given in Table 2. In the original data, raised risks of childhood cancers were greatest among children whose mothers left employment before the child's conception and, for all childhood cancers combined, this risk was significantly raised (RR=5.50, 95% CI: 1.20, 51). However, in the new data, the RRs are not significantly raised for any of the exposure periods of maternal employment. This analysis is described in more detail in the HPA report (Muirhead *et al*, 2009).

## Discussion

### Study characteristics

This study is based entirely on data from existing registers and thus avoids potential bias arising from the selection of cases and controls or as a result of differential response rates. In line with current legislation and good practice, women radiation workers were advised of their right to withdraw from the study, but only 9 out of 15 849 chose to do so. The great majority of UK radiation workers in the nuclear industry are included in the NRRW, as are (with the exception of those working in the medical field) a good proportion of radiation workers employed elsewhere in the United Kingdom. A significant minority of these employees has been female, many of whom have been occupationally exposed for only short periods of time. An advantage of our study design is that all employees, regardless of the length of time for which they were exposed, contribute to the analysis.

No information is available that would enable predictions to be made of the number of matches to be expected. Because of the importance of identifying every mother, whether of a case or control child, who was an NRRW member, great attention was paid to every possible link. The researchers were blind to the case/control status of possible matches; thus if any genuine matches were not identified, they were equally likely to have involved the mothers of case or control children. The linkage would have been simpler and less time consuming if information on the mother's date of birth were available from the child's birth registration details, but there is a legally binding embargo on this extra information. However, we feel that despite this the record linkage procedures used here were generally successful.

The inclusion criteria for both childhood cancer cases and women NRRW members were the same for the two studies. However, although there is no clear boundary to the timing of exposure for the matched NRRW women in the two studies, women in the new study tend to have been exposed more recently, when mean annual doses were lower (Muirhead *et al*, 2009, Table A6).

### Comparison of main results from the two studies

In the original Record Linkage Study (Draper *et al*, 1997a, 1997b) the risk of childhood cancer in the offspring of female radiation workers was statistically significantly greater than that among the offspring of non-radiation workers. The main motivation for conducting this new study was to determine whether these elevated risks were maintained in later data. The new data do not support the association seen earlier. Indeed, the tests for heterogeneity revealed a significant difference (*χ*^2^ on 1 d.f.=4.75, *P*=0.03) in the RRs for childhood cancer overall between the new data and the original data, suggesting that we should interpret results taken from the pooled dataset with caution.

### Effect of employment timing

In the original study (Draper *et al*, 1997a, 1997b) there was a statistically significant excess of LNHL in the offspring of male radiation workers. However, a subsequent comparison between the offspring of men who had left radiation work before conception and those still employed on the date of conception showed that this excess was concentrated in the latter group (Sorahan *et al*, 2003). This was consistent with the idea that any causative factor was one that operated among the population around a nuclear site, rather than continuing to affect a worker when he left (as might be expected of unrepaired radiation-induced germ cell damage). This supported the suggestion by Kinlen (1988) that population mixing might be largely responsible for the observed increase in childhood leukaemia. In isolated locations, herd immunity to a postulated virus infection would tend to be low, giving conditions conducive to epidemics following a population influx; in such situations elevated levels of childhood leukaemia might occur as a rare response to virus infection.

The numbers of cases are too small to throw any useful light on whether a similar effect might be seen in the offspring of female radiation workers (Table 2). It is very likely that any infective mechanism operating in the area of nuclear installations would affect children whether their parents worked at the installation or not. Thus somewhat elevated levels of LNHL may be found in the offspring of female radiation workers for this reason. However, women leaving nuclear industry employment may be more likely than men to remain living in the vicinity; in this case the leukaemia risks for their offspring would not be related to patterns of employment.

Possible explanations of the raised risk found earlier among children of women radiation workers are that it was due either to chance or to some other aetiological factor. The possibility that the finding was simply because of chance is strengthened by the fact that the tumours observed in the offspring of women radiation workers are in different diagnostic categories. However, it is possible that some small effect of population mixing may be taking place. There is good evidence that childhood leukaemia is related to exposure to infection (McNally and Eden, 2004) and some indirect evidence that this might also be true for other childhood cancers. Although the studies of Kinlen and colleagues (Kinlen, 1988, 1995, 1997; Kinlen *et al*, 1993) do not consider other childhood cancers, analyses carried out for the eleventh COMARE report (2006) found that incidence rates for some other childhood cancers were related to levels of measures of socioeconomic status, which may in turn be related to exposure to infections.

### *In utero* exposure

It is now widely accepted that very low exposures to X-rays *in utero* can cause leukaemia and other childhood cancers. Wakeford and Little (2003) estimate that the excess RR of childhood cancer resulting from *in utero* exposure may be around 50 per Gy, that is, 0.05 per mGy (for X-rays, 1 mGy is taken to be equivalent to 1 mSv). They remark that there is reason to believe that this could be an overestimate. The doses involved probably overlap with those experienced by workers in this study, but there are various differences in the nature of the exposure to radiation: the doses in the studies of medical radiation were delivered by one or a few instantaneous exposures, usually during the third trimester, whereas those in this study were probably usually delivered over a period of weeks or months. Moreover, they were, on average, less than the total dose from natural radiation during pregnancy, which can be estimated to be around 0.8 mSv (Simmonds *et al*, 1995). It is difficult to reconcile our estimated relative risk of 7.0 with the results from the diagnostic radiation studies. In view of the small numbers involved, and the borderline significance of our estimate (*P*=0.07), this result may well be due to chance.

Comparison of the original and new data sets suggests that in the earlier years, women tended to work for a shorter period of time, finishing work some time before the child in question was born and frequently not returning to work, whereas in more recent times, women are in employment, often as monitored workers, for most of the period before childbirth, and frequently return to work subsequently. In the original study only a few of the NRRW women identified as case or control mothers (4 out of 18) were exposed while pregnant, compared with 4 out of 11 in the new data. If more women are indeed continuing in radiation work while pregnant, it is important that accurate estimates of any possible cancer risk incurred by their subsequent children are available.

### Comparison with other studies

Published studies considering parental preconception irradiation predating our original study (Draper *et al*, 1997a) were reviewed at the time of publication of that study. Since then, the Nuclear Industry Family Study (Roman *et al*, 1999) (NIFS), an interview study, has reported on several aspects of the health of nuclear workers and their families but found no significant excess cancer risk for the children of exposed women workers.

Some exposed occupational groups that normally include a higher proportion of women are not covered by the NRRW. One such group is medical radiographers; however, a study of this group (Roman *et al*, 1996) found no excess cancer in the offspring of the approximately 5000 women workers. Likewise, a recent large scale study of childhood cancer in the offspring of US radiologic technologists showed no convincing evidence of increased risk in the children of women workers (Johnson *et al*, 2008).

An American report on areas around three nuclear facilities in the United States (Sever *et al*, 1997) studied the association between parental exposure to ionising radiation and childhood cancer. Although intended primarily to replicate earlier studies of men (Gardner *et al*, 1990a, 1990b; McLaughlin *et al*, 1993), the study also examined the risk of maternal preconception exposure for various cancer diagnostic subgroups. No significantly raised cancer risks were reported in children whose mothers were exposed before conception or during pregnancy.

No evidence of an increased risk has been found in other studies of malignant disease in the offspring of parents exposed to radiation; indeed a recent international workshop concluded that ‘no human germ-cell mutagen has been confirmed to date’ (Wyrobek *et al*, 2007).

In conclusion, the new data provide no evidence of an increased risk of childhood cancer associated with maternal preconception radiation work and thus, do not support our earlier finding of a raised risk in the offspring of female radiation workers. Considering the pooled data, a weak association was found between maternal radiation work during pregnancy and childhood cancer in offspring, although the evidence is limited by the small numbers of linked cases and controls.

## Figures and Tables

**Table 1:** Relative risks for childhood cancer by mother's radiation dose before child's conception and while pregnant

		**Original data^a^**			**New data**			**Pooled data**		
	**Dose group (mSv)**	**No. of cases**	**No. of controls**	**Relative risk (95% CI)^b^**	**No. of cases**	**No. of controls**	**Relative risk (95% CI)^b^**	**No. of cases**	**No. of controls**	**Relative risk (95% CI)^b^**
*Leukaemia and NHL*										
Non-radiation worker^c^		13 855	13 858	1.0	6206	6204	1.0	20 061	20 062	1.0
Total preconception dose										
	<0.1^d^	0	0	—	0	0	—	0	0	—
	0.1–4.9	3	1	3.00 (0.24, 157)	1	3	0.33 (0.01, 4.2)	4	4	1.00 (0.19, 5.37)
	5.0–49.9	0	0	—	1	1	1.00 (0.01, 79)	1	1	1.00 (0.01, 79)
	50.0+	1	0	1.00 (0.03, Inf)^e^	0	0	—	1	0	1.00 (0.03, Inf)^e^
All preconception dose levels combined		4	1	4.00 (0.40, 197)	2	4	0.50 (0.04, 3.49)	6	5	1.20 (0.31, 4.97)
Radiation worker, no *in utero* employment		4	1	4.00 (0.40, 197)	0	3	0.26 (0.00, 2.42)^e^	4	4	1.00 (0.19, 5.37)
Radiation worker, *in utero* dose^f^	<0.1^d^	0	0	—	0	0	—	0	0	—
	0.1–0.9	0	0	—	2	1	2.00 (0.10, 118)	2	1	2.00 (0.10, 118)
	1.0–1.9	0	0	—	0	0	—	0	0	—
	2.0+	0	0	—	0	0	—	0	0	—
All *in utero* dose levels combined		0	0	—	2	1	2.00 (0.10, 118)	2	1	2.00 (0.10, 118)
										
Monitored for internal exposure										
Radiation worker, not monitored^g^		4	1	4.00 (0.40, 197)	0	4	0.19 (0.00, 1.52)^e^	4	5	0.80 (0.16, 3.72)
Radiation worker, monitored^g^		0	0	—	2	0	2.41 (0.19, Inf)^e^	2	0	2.41 (0.19, Inf)^e^
Radiation worker, monitored *vs* non monitored^g^				—			8.69 (0.51, Inf)^e^			2.29 (0.16, Inf)^e^
										
*All cancers excluding leukaemia and NHL*										
Non-radiation worker^c^		21 778	21 787	1.0	10 754	10 753	1.0	32 532	32 540	1.0
Total preconception dose										
	<0.1^d^	2	0	2.41 (0.19, Inf)^e^	0	0	—	2	0	2.41 (0.19, Inf)^e^
	0.1–4.9	5	1	5.00 (0.56, 237)	2	3	0.67 (0.06, 5.82)	7	4	1.75 (0.44, 8.15)
	5.0–49.9	2	1	2.00 (0.10, 118)	0	0	—	2	1	2.00 (0.10, 118)
	50.0+	2	0	2.41 (0.19, Inf)^e^	0	0	—	2	0	2.41 (0.19, Inf)^e^
All preconception levels combined		11	2	5.50 (1.20, 51)^h^	2	3	0.67 (0.06, 5.82)	13	5	2.60 (0.87, 9.32)
Radiation worker, no *in utero* employment		7	2	3.50 (0.67, 35)	1	3	0.33 (0.01, 4.15)	8	5	1.60 (0.46, 6.22)
Radiation worker, *in utero* dose^f^	<0.1^d^	2	0	2.41 (0.19, Inf)^e^	0	0	—	2	0	2.41 (0.19, Inf)^e^
	0.1–0.9	1	0	1.00 (0.03, Inf)^e^	1	0	1.00 (0.03, Inf)^e^	2	0	2.41 (0.19, Inf)^e^
	1.0–1.9	0	0	—	0	0	—	0	0	—
	2.0+	1	0	1.00 (0.03, Inf)^e^	0	0	—	1	0	1.00 (0.03, Inf)^e^
All *in utero* dose levels combined		4	0	5.29 (0.66, Inf)^e^	1	0	1.00 (0.03, Inf)^e^	5	0	6.73 (0.92, Inf)^e^e,i
Monitored for internal exposure										
Radiation worker, not monitored^g^		11	1	11.0 (1.60, 473)^h^	1	2	0.50 (0.01, 9.60)	12	3	4.00 (1.08, 22)^h^
Radiation worker, monitored^g^		0	1	1.00 (0.00, 39)^e^	1	1	1.00 (0.01, 79)	1	2	0.50 (0.01, 9.60)
Radiation worker, monitored *vs* non monitored^g^				0.18 (0.00, 7.09)^e^			1.73 (0.01, 234)			0.14 (0.002, 3.63)
										
*All childhood cancers*										
Non-radiation worker^c^		35 633	35 645	1.0	16 960	16 957	1.0	52 593	52 602	1.0
Total preconception dose										
	<0.1^d^	2	0	2.41 (0.19, Inf)^e^	0	0	—	2	0	2.41 (0.19, Inf)^e^
	0.1–4.9	8	2	4.00 (0.80, 37)	3	6	0.50 (0.08, 2.34)	11	8	1.38 (0.50, 3.94)
	5.0–49.9	2	1	2.00 (0.10, 118)	1	1	1.00 (0.01, 79)	3	2	1.50 (0.17, 18)
	50.0+	3	0	3.85 (0.41, Inf)^e^	0	0	—	3	0	3.85 (0.41, Inf)^e^
All preconception dose levels combined		15	3	5.00 (1.42, 27)^h^	4	7	0.57 (0.12, 2.25)	19	10	1.90 (0.84, 4.58)
Radiation worker, no *in utero* employment		11	3	3.67 (0.97, 20)	1	6	0.17 (0.004, 1.37)	12	9	1.33 (0.52, 3.58)
Radiation worker, *in utero* dose^f^	<0.1^d^	2	0	2.41 (0.19, Inf)^e^	0	0	—	2	0	2.41 (0.19, Inf)^e^
	0.1–0.9	1	0	1.00 (0.03, Inf)^e^	3	1	3.00 (0.24, 157)	4	1	4.00 (0.40, 197)
	1.0–1.9	0	0	—	0	0	—	0	0	—
	2.0+	1	0	1.00 (0.03, Inf)^e^	0	0	—	1	0	1.00 (0.03, Inf)^e^
All *in utero* dose levels combined		4	0	5.29 (0.66, Inf)^e^	3	1	3.00 (0.24, 157)	7	1	7.00 (0.90, 315)^j^
Monitored for internal exposure										
Radiation worker, not monitored^g^		15	2	7.50 (1.74, 68)^h^	1	6	0.17 (0.004, 1.37)	16	8	2.00 (0.81, 5.40)
Radiation worker, monitored^g^		0	1	1.00 (0.00, 39)^e^	3	1	3.00 (0.24, 157)	3	2	1.50 (0.17, 18)
Radiation worker, monitored *vs* not monitored^g^				0.20 (0.00, 7.80)^e^			12.00 (0.50, 1097)			0.76 (0.07, 11)

a Using the corrected doses described by Sorahan and colleagues (Sorahan *et al*., 2003) which superseded the values reported by Draper and colleagues (Draper *et al*, 1997a, 1997b).

b Exact 95% CI, calculated using LogXact (2005).

c Not known to have been monitored for occupational exposure before the conception of the survey child. All relative risks are calculated using this as the reference group.

d Includes monitored workers whose dose, after correction, is zero (see footnote a).

e Conditional maximum-likelihood estimate is not available because the sufficient statistic is at one extreme of its range. The median unbiased point estimate is shown with 95% confidence interval (CI), see Appendix B1 of Muirhead *et al*, (2009) for a fuller description of the statistical methodology.

f *In utero* doses were obtained only for women who were monitored before conception.

g Monitoring status refers to internal radiation exposure. All of these radiation workers were monitored for external exposure.

h *P*<0.05.

i *P*=0.06.

j *P*=0.07.

**Table 2:** Relative risks for childhood cancer by time of maternal employment at facilities participating in the NRRW

	**Original data**			**New data**			**Pooled data**		
**Variable with levels**	**Cases**	**Controls**	**Relative risk (95% CI)^a^**	**Cases**	**Controls**	**Relative risk (95% CI)^a^**	**Cases**	**Controls**	**Relative risk (95% CI)^a^**
*Leukaemia and NHL*									
Left employment before conception and had no subsequent employment^b^									
No	13 855	13 858	1.0	6208	6205	1.0	20 063	20 063	1.0
Yes	4	1	4.00 (0.40, 197)	0	3	0.26 (0.00, 2.42)^c^	4	4	1.00 (0.19, 5.37)
Still in employment at conception or resumed employment subsequently^b^									
No	13 859	13 859	1.0	6206	6207	1.0	20 065	20 066	1.0
Yes	0	0	—	2	1	2.00 (0.10, 118)	2	1	2.00 (0.10, 118)
Still in employment during year of diagnosisb,^d^									
No	13 859	13 859	1.0	6207	6 208	1.0	20 066	20 067	1.0
Yes	0	0	—	1	0	1.00 (0.03, inf)^c^	1	0	1.00 (0.03, inf)^c^
*All cancers other than leukaemia and NHL*									
Left employment before conception and had no subsequent employment^b^									
No	21 782	21 788	1.0	10 755	10 754	1.0	32 537	32 542	1.0
Yes	7	1	7.00 (0.90, 315)^e^	1	2	0.50 (0.01, 9.60)	8	3	2.67 (0.64, 16)
Still in employment at conception or resumed employment subsequently^b^									
No	21 785	21 788	1.0	10 755	10 755	1.0	32 540	32 543	1.0
Yes	4	1	4.00 (0.40, 197)	1	1	1.00 (0.01, 79)	5	2	2.50 (0.40, 26)
Still in employment during year of diagnosisb,^d^									
No	21 788	21 788	1.0	10 755	10 755	1.0	32 543	32 543	1.0
Yes	1	1	1.00 (0.01, 79)	1	1	1.00 (0.01, 79)	2	2	1.00 (0.07, 14)
*All childhood cancers*									
Left employment before conception and had no subsequent employment^b^									
No	35 637	35 646	1.0	16 963	16 959	1.0	52 600	52 605	1.0
Yes	11	2	5.50 (1.20, 51)^f^	1	5	0.20 (0.004, 1.79)	12	7	1.71 (0.62, 5.14)
Still in employment at conception or resumed employment subsequently^b^									
No	35 644	35 647	1.0	16 961	16 962	1.0	52 605	52 609	1.0
Yes	4	1	4.00 (0.40, 197)	3	2	1.50 (0.17, 18)	7	3	2.33 (0.53, 14)
Still in employment during year of diagnosisb,^d^									
No	35 647	35 647	1.0	16 962	16 963	1.0	52 609	52 610	1.0
Yes	1	1	1.00 (0.01, 79)	2	1	2.00 (0.10, 118)	3	2	1.50 (0.17, 18)

a Exact 95% CI, calculated using LogXact (2005).

b With a non-zero preconceptional radiation dose recorded with NRRW.

c Conditional maximum-likelihood estimate is not available because the sufficient statistic is at one extreme of its range. The median unbiased point estimate is shown with 95% confidence interval (CI), see Appendix B1 of Muirhead *et al* (2009) for a fuller description of the statistical methodology.

d Refers to 1st January in the year in which the case child was diagnosed.

e *P*=0.07.

f *P*<0.05.
